# The impact of low-carbon city pilot policies on urban energy intensity

**DOI:** 10.3389/fpubh.2026.1806792

**Published:** 2026-05-15

**Authors:** Yuren Qian, Jiahan Hu, Bingnan Guo, Tangfa Liu, Hao Hu

**Affiliations:** 1 School of Humanities and Social Sciences, Jiangsu University of Science and Technology, Zhenjiang, China; 2 College of Engineering, University of Perpetual Help System Laguna, Laguna, Philippines; 3 School of Economics and Management, Gannan University of Science and Technology, Ganzhou, China; 4 School of Economics, Shanghai University, Shanghai, China

**Keywords:** causal inference, fiscal support, green technological innovation, low-carbon city pilot policy, urban energy intensity

## Abstract

Urban energy intensity measures the energy required per unit of economic output and is closely linked to energy-related emissions that shape air quality and climate-related health risks. Using panel data from 273 prefecture-level Chinese cities (2006–2021), we estimate the causal impact of China's Low-Carbon City Pilot Scheme (LCCPS) on urban energy intensity. We apply a partially linear double/debiased machine learning framework with city and year fixed effects, enabling flexible adjustment for high-dimensional confounders. The LCCPS lowers urban energy intensity by about 0.11 units, and the result is robust to alternative specifications. Channel analyses suggest that pilot designation strengthens fiscal support intensity and stimulates enterprise green innovation, which together contribute to reduced energy intensity. Effects are strongest in large and coastal cities and in resource-scarce regions, but weaker in small and medium-sized cities, inland areas, and resource-rich regions. By reducing the energy required for economic activity, low-carbon pilots may also generate public-health co-benefits through cleaner urban environments.

## 1 Introduction

Rapid urbanization concentrates population, industry, and transport in cities, making urban areas central to both climate mitigation and environmental health protection (1). Energy use in urban economies drives greenhouse-gas emissions and local air pollution, with established impacts on respiratory and cardiovascular health and other outcomes (2). In China, the carbon peaking and carbon neutrality agenda (“dual carbon” goals) has pushed urban energy governance to the forefront of national strategy (3). Because cities account for a large share of energy consumption and emissions, improving urban energy efficiency is essential for sustainable development and for reducing health-damaging environmental exposures (4).

Urban energy intensity (UEI), commonly defined as total energy consumption per unit of gross regional product, captures how energy-dependent local economic activity is (5). Lower UEI indicates that a city can generate output with less energy input—often through cleaner energy mixes, technological upgrading, and structural change (6). From a public health perspective, sustained UEI reductions can help lower energy-related emissions per unit of activity, supporting cleaner air and healthier urban living environments (7). Yet cities may be locked into energy-intensive growth models, and firms can underinvest in low-carbon technology because of high upfront costs and uncertainty (8, 9).

China's Low-Carbon City Pilot Scheme (LCCPS) is a flagship, place-based policy intended to overcome these constraints and accelerate urban decarbonization (10). Initiated by the National Development and Reform Commission, pilots were announced in three waves (2010, 2012, and 2017), expanding from early adopters to a broader set of prefecture-level cities and selected districts/counties (11). Pilot jurisdictions are required to integrate energy-saving and carbon-reduction objectives into development planning and to pursue locally tailored actions such as energy-structure adjustment, industrial upgrading, low-carbon urban planning, and policy innovation (12–14). This institutional setting offers a useful quasi-experimental context for evaluating whether urban low-carbon governance can improve energy efficiency with potential downstream health co-benefits (15).

Prior empirical work generally reports that the LCCPS improves energy efficiency and reduces energy intensity, most often using difference-in-differences and matching-based approaches (16–18). However, two issues remain: (i) conventional specifications may inadequately capture nonlinearities and high-dimensional confounding, leading to potential misspecification; and (ii) the distribution of benefits across city types is not fully understood, limiting discussion of equity-relevant implications for environmental and health conditions.

We address these issues by estimating the causal effect of the LCCPS on UEI with a double machine learning (DML) framework that combines econometric identification with flexible machine-learning nuisance estimation. We then examine two pathways highlighted in the policy design—fiscal support and enterprise green innovation—and explore heterogeneity by city size, coastal location, and resource endowment using grouped DML and causal forests. By positioning UEI as an upstream determinant of emissions and urban environmental quality, the study provides evidence relevant to both decarbonization policy and public-health co-benefits.

## 2 Policy background and research hypotheses

### 2.1 Policy background

**Figure 1:**
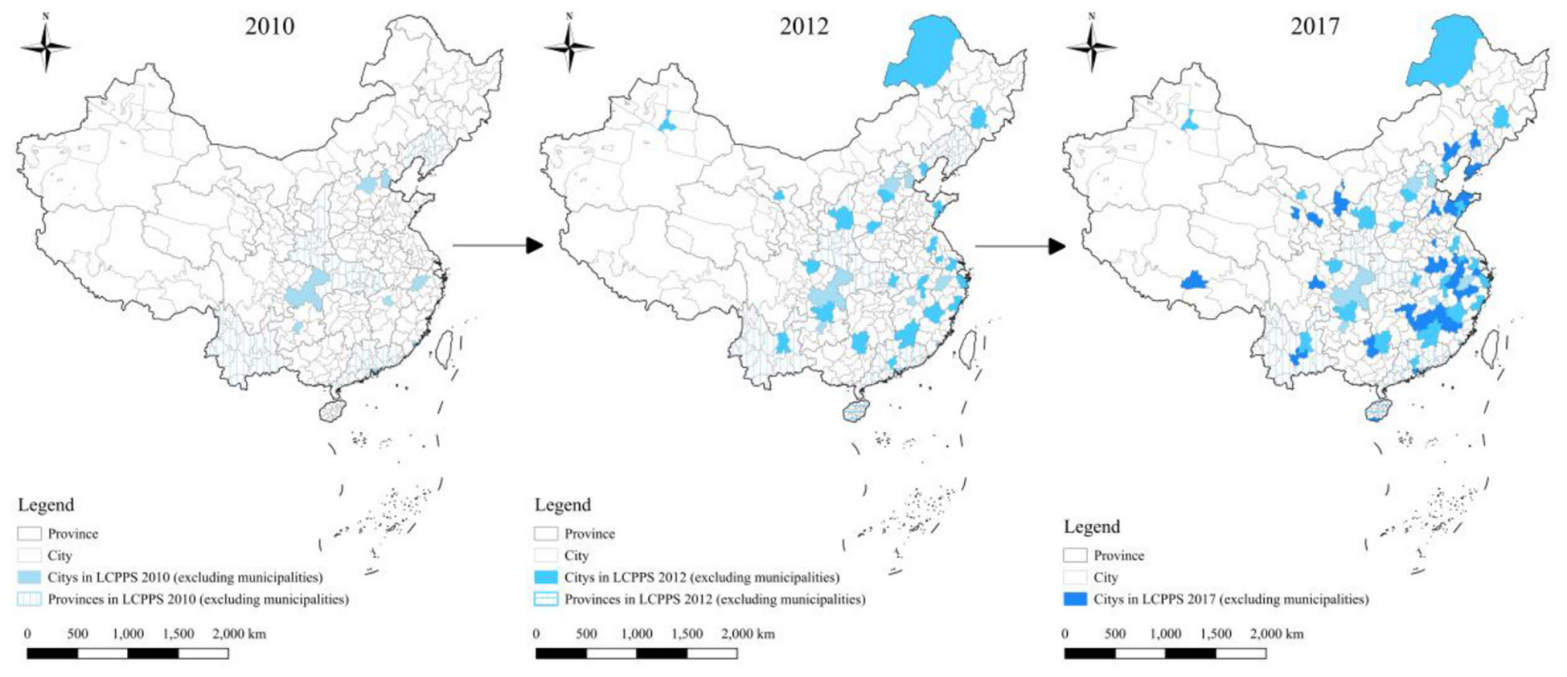
The distribution of the three batches of LCCPS areas.

Cities are major hubs of economic activity and energy use; lowering their energy intensity is therefore important for China's “dual carbon” objectives and for high-quality development (19, 20). Low-carbon city construction aims to decouple growth from energy consumption by improving energy efficiency, upgrading industrial structures, and encouraging cleaner technologies (21–23). The National Development and Reform Commission launched the LCCPS in three batches (2010, 2012, 2017), covering pilot provinces/municipalities and cities and progressively strengthening requirements—from emissions data systems, to target responsibility, to assessment and energy-consumption monitoring frameworks (24–26). A distinctive feature of the LCCPS is its implementation flexibility: local governments are granted discretion to design measures aligned with local development stages and energy-use profiles, using a mix of regulation and incentives to guide firms and residents toward lower-carbon production and consumption (27). Figure 1 maps the pilot areas. Table 1 presents the number of cities implementing the LCCPS policy in each year.

**Table 1:** LCCPS areas.

Year	Areas
2010	8 pilot cities and 5 pilot provinces/regions (first batch)
2012	27 pilot cities and 3 provincial-level pilots (second batch)
2017	45 pilot cities and 4 districts/counties (third batch)

### 2.2 Research hypotheses

This study examines whether the LCCPS reduces UEI and investigates two candidate pathways emphasized in the policy toolkit: (i) enterprise green technological innovation and (ii) government fiscal support. In principle, a comprehensive low-carbon institutional framework can promote energy-structure optimization and technology upgrading, thereby reducing energy use per unit of output. Consistent with evidence that low-carbon city construction can improve energy efficiency and support urban green transformation (28), and drawing on the Porter Hypothesis and institutional innovation theory (29, 30), we posit an overall UEI-reducing effect of the pilots (31).

**Hypothesis 1**: The LCCPS significantly reduces UEI.

At the firm level, the LCCPS may lower UEI by stimulating green innovation. Policy instruments such as special funds, tax incentives, and subsidies can reduce the cost and risk of R&D, encouraging firms to develop and adopt cleaner energy technologies and energy-saving processes (32). Related work suggests that pilots can facilitate equipment upgrading (e.g., energy-saving capital deepening) and accelerate digital transformation that improves energy management (33, 34). As innovation raises productivity and can reduce energy use per unit of output (35), green technological innovation is a plausible transmission channel. Empirical evidence also indicates that innovation can account for a substantial share of the pilot effect on energy intensity (36).

**Hypothesis 2**: The LCCPS reduces UEI by promoting enterprise green technological innovation.

At the city level, the LCCPS may also operate through fiscal support. Low-carbon policies often involve higher environmental and green-investment spending, improved monitoring capacity, and tax or subsidy programs that steer resources toward low-energy and low-carbon activities (37). Fiscal incentives can speed industrial restructuring by supporting clean industries and phasing out inefficient capacity (38). In addition, pilots typically strengthen governance arrangements for dual control of total energy use and intensity, and may encourage market-based tools such as carbon trading to motivate efficiency improvements in firms (39). These mechanisms can also be understood within the framework of the Environmental Kuznets Curve (EKC), which suggests that as economic development progresses, improvements in technology and structural transformation may contribute to reductions in energy intensity. From this perspective, policy-induced investments and institutional enhancements under the LCCPS can facilitate technological upgrading and reallocation of resources toward less energy-intensive activities, thereby reinforcing the declining stage of the EKC relationship. This interpretation is consistent with recent studies that extend the EKC framework to energy intensity (40, 41).

**Hypothesis 3**: The LCCPS reduces UEI by strengthening fiscal support for low-carbon development.

## 3 Research design

### 3.1 Model specification

To estimate the causal effect of the LCCPS on UEI while allowing for high-dimensional controls and nonlinear relationships, we use a double machine learning (DML) approach. DML combines causal identification with machine-learning prediction for nuisance components and uses orthogonalization and cross-fitting to reduce bias from overfitting and model misspecification (42). We focus on the partially linear DML model, which is widely used for policy evaluation. Let UEI denote urban energy intensity, Event indicate LCCPS pilot status, and X represent a potentially high-dimensional set of controls. The model allows flexible (possibly nonlinear) functions linking X to both UEI and treatment assignment, while identifying the average causal effect of the policy under standard assumptions:


$$\begin{array}{l} UEI_{i}=\theta_{0}Event_{i}+g\left(X_{i}\right)+U_{i} \end{array}$$ (1)

$$\begin{array}{l} Event_{i}=m\left(X_{i}\right)+V_{i} \end{array}$$ (2)

In this setting, UEI is the outcome variable; Event equals 1 if a city is included in the LCCPS in year t and 0 otherwise; X includes observed covariates that may affect both UEI and policy assignment; g (·) and m (·) are unknown functions; and the disturbance terms capture unobserved factors. The parameter θ is the policy effect of interest. The specific steps are as follows:

While fully utilizing the predictive capabilities of machine learning, it is still necessary to ensure robust inference of the main parameters. To this end, Neyman's orthogonalization idea is introduced by constructing the following “moment function”:


$$\begin{array}{r} \psi_{i}(\theta ,g,m)=\left(D_{i}-m\left(X_{i}\right)\right)[\left(UEI_{i}-g\left(X_{i}\right)\right)\\ -\theta \left(D_{i}-m\left(X_{i}\right)\right)] \end{array}$$ (3)

If θ = θ_0_, and machine learning approximates *g*(*X*_*i*_),*m*(*X*_*i*_)sufficiently accurately, then:


$$\begin{array}{l} E\left[\psi \left(\theta ,g\left(X_{i}\right)\right)\right]=0 \end{array}$$ (4)

Expanding Equation 4, we get:


$$\begin{array}{l} E\left[D_{i}-m\left(X_{i}\right)\right]\left(UEI_{i}-g\left(X_{i}\right)\right)-\theta_{0}E{\left[D_{i}-m\left(X_{i}\right)\right]}^{2}=0 \end{array}$$ (5)

Solving for this gives:


$$\begin{array}{l} \theta_{0}=\frac{E\left[D_{i}-m\left(X_{i}\right)\right]\;\left[UEI_{i}-g\left(X_{i}\right)\right]\;}{E{\left[D_{i}-m\left(X_{i}\right)\right]}^{2}} \end{array}$$ (6)

Replacing expectation operations with sample averages and using machine learning to estimate function values:


$$\begin{array}{l} \hat{g}\left(X_{i}\right)\approx g\left(X_{i}\right),\;\hat{m}\left(X_{i}\right)\approx m\left(X_{i}\right) \end{array}$$ (7)

The regularized estimator for θ_0_ is thus:


$$\begin{array}{c} {\hat{\theta }}_{0}={\left[\frac{1}{N}\overset{N}{\underset{i=1}{\sum }}{\left(D_{i}-\hat{m}\left(X_{i}\right)\right)}^{2}\right]}^{-1}\cdot \\ \left[\frac{1}{N}\overset{N}{\underset{i=1}{\sum }}\left(D_{i}-\hat{m}\left(X_{i}\right)\right)\left(UEI_{i}-\hat{g}\left(X_{i}\right)\right)\right] \end{array}$$ (8)

Referencing Chernozhukov's research, it is emphasized that Neyman orthogonalization can only ensure that the regularized bias product term converges faster than *N*^−1/4^ (a rate typically guaranteed by sparsity in regressions like Lasso), thereby avoiding bias from high-order estimation error product terms. However, if machine learning training and residual regression for the target parameter residual regression are performed on the same sample batch, a so-called “third term” bias (the product of prediction error terms and estimation error terms) will inevitably emerge.

Specifically, subtracting $\hat{g}\left(X_{i}\right)$ from both sides of Equation 1, we have:


$$\begin{array}{l} UEI_{i}-\hat{g}\left(X_{i}\right)=\theta_{0}D_{i}+g\left(X_{i}\right)-\hat{g}\left(X_{i}\right)+U_{i} \end{array}$$ (9)

Substituting Equations 2 and 9 into Equation 8, we find that we can decompose


$$\begin{array}{l} \frac{1}{\sqrt{N}}\overset{N}{\underset{i=1}{\sum }}V_{i}\left({UEI}_{i}-\hat{g}\left(X_{i}\right)\right) \end{array}$$ (10)

into three parts:

a. The product of two error terms, specifically:


$$\begin{array}{l} \frac{1}{\sqrt{N}}\overset{N}{\underset{i=1}{\sum }}U_{i}V_{i}\overset{P}{\sim }N\left(0,\sigma_{UV}^{2}\right) \end{array}$$ (11)

b. The regularized bias product term, namely:


$$\begin{array}{l} \frac{1}{\sqrt{N}}\overset{N}{\underset{i=1}{\sum }}\left(\hat{g}\left(X_{i}\right)-g\left(X_{i}\right)\right)\left(\hat{m}\left(X_{i}\right)-m\left(X_{i}\right)\right) \end{array}$$ (12)

Even if the convergence rates of both estimation errors are relatively slow, their product in regularization will converge faster than *N*^−1/4^, thus becoming an infinitesimal term as *N* → ∞.

c. The product of error terms and estimation errors, specifically:


$$\begin{array}{l} \frac{1}{\sqrt{N}}\overset{N}{\underset{i=1}{\sum }}V_{i}\left(\hat{g}\left(X_{i}\right)-g\left(X_{i}\right)\right),\frac{1}{\sqrt{N}}\overset{N}{\underset{i=1}{\sum }}U_{i}\left(\hat{m}\left(X_{i}\right)-m\left(X_{i}\right)\right) \end{array}$$ (13)

Assumption:


$$\begin{array}{l} \hat{g}\left(X_{i}\right)=g\left(X_{i}\right)+\frac{U_{i}}{N^{\frac{1}{2}-\delta }} \end{array}$$ (14)

Therefore:


$$\begin{array}{l} \hat{g}\left(X_{i}\right)-g\left(X_{i}\right)=O_{p}\left(N^{-\frac{1}{2}+\bar{\delta }}\right) \end{array}$$ (15)

This is a relatively fast convergence rate for an almost parametric model. Substituting this into (13), we have:


$$\begin{array}{r} \frac{1}{\sqrt{N}}\underset{i}{\sum }V_{i}\left(\hat{g}\left(X_{i}\right)-g\left(X_{i}\right)\right)=\sqrt{N}O_{p}\left(N^{-\frac{1}{2}+\bar{\delta }}\right)\\ =O_{p}\left(N^{\bar{\delta }}\right)\to \infty \end{array}$$ (16)

The final result $O_{p}\left(N^{\bar{\delta }}\right)$ is a quantity that asymptotically diverges as the sample size *N* increases, which means the estimation bias does not disappear but instead continues to increase with increasing sample size. This bias primarily stems from the fact that the sample used to estimate $\hat{m}\left(\cdot \right)$ contains information that is potentially correlated with *V*_*i*_, making *V*_*i*_ and $\hat{g}\left(X_{i}\right)-g\left(X_{i}\right)$ potentially correlated. In other words, because the same sample is used both to simultaneously participate in estimating $\hat{g}\left(\cdot \right)$ and in estimating $\hat{g}$'s θ_0_, this leads to non-negligible estimation bias.

To eliminate this bias, this paper adopts a cross-fitting method. The specific steps are as follows:

(1) Randomly divide the sample into S parts: *I*_1_, *I*_2_, …, *I*_*S*_;
(2) Estimate machine learning models $\hat{m}$ and $\hat{g}$ on the complement of the s-th sample;
(3) Calculate residuals using the s-th sample;
(4) Repeat the above steps for each sample part;
(5) Finally, take a weighted average of the estimates from each part to obtain the final ${\hat{\theta }}_{0}$.

Through this approach, the training of machine learning models and the estimation of parameters are conducted on different samples, effectively avoiding the “third term” bias problem. This method not only ensures unbiased estimation but also enhances estimation efficiency.

### 3.2 Variable definitions

#### 3.2.1 Dependent variable

The outcome variable is urban energy intensity (UEI). Following Yang & Wei (43), we measure UEI as total energy consumption divided by gross regional product. Lower values indicate that a city produces the same economic output with less energy input, consistent with higher energy-use efficiency.

#### 3.2.2 Core independent variable

The key explanatory variable is LCCPS pilot status (Event). We code Event as 1 for city–year observations covered by the 2010, 2012, or 2017 pilot lists announced by the National Development and Reform Commission, and 0 otherwise. The policy timing is aligned with the official circulars for each batch.

#### 3.2.3 Control variables

DML can flexibly select among a rich set of pre-specified covariates. To reduce omitted-variable bias, we include controls that are plausibly related to UEI and to pilot selection, capturing economic conditions, urban form, infrastructure, and openness:

- ① Economic development (Gdp): ln (GRP). More developed cities may consume more energy in total but can also achieve lower UEI through technology and structural upgrading.
- ② Population density (Density): ln (population). Agglomeration can facilitate knowledge spillovers and efficiency gains, potentially lowering UEI.
- ③ Internet development (Internet): ln (internet users per 10,000 people). Digitalization can improve information flows and energy management, affecting UEI through production and lifestyle channels.
- ④ Transport development (Transport): ln (road passenger volume). Transport scale may influence energy demand and the spatial organization of economic activity.
- ⑤ Industrial structure (Structure): ratio of secondary to tertiary value-added. Structural upgrading toward services is typically associated with lower energy intensity.
- ⑥ Infrastructure (Infra): ln (per-capita road area). Infrastructure can raise energy use during construction but may improve long-run efficiency via connectivity.
- ⑦ Trade openness (Fdi): (imports + exports)/GRP. Openness can shift industrial composition and facilitate technology diffusion, with ambiguous implications for UEI.
- ⑧ Education level (Edu): ln(university students per 10,000 people). Human capital may strengthen innovation and environmental governance capacity, contributing to lower UEI.

To allow for nonlinearities, we include quadratic terms for continuous controls following Li et al. (44). All specifications include city and year fixed effects to absorb time-invariant city characteristics and common shocks.

### 3.3 Sample selection and data sources

We construct an unbalanced panel of 273 prefecture-level and above Chinese cities from 2006 to 2021. Cities with extensive missing observations are excluded; for variables with limited gaps, we apply linear interpolation. Data come primarily from the China Urban Statistical Yearbook and China Energy Statistical Yearbook, supplemented with green patent information from the China National Intellectual Property Administration and WIPO lists, and municipal statistical yearbooks. Continuous variables are winsorized at the 1% tails to limit outlier influence; descriptive statistics are reported in Table 2.

**Table 2:** Descriptive statistics.

Var	Obs	Mean	SD	Min	Max
*ECI*	4,330	0.942	0.598	0.069	8.063
*Event*	4,330	0.239	0.427	0.000	1.000
*Gdp*	4,330	16.385	1.012	13.461	19.884
*Density*	4,330	5.758	0.914	1.386	7.882
*Internet*	4,330	0.197	0.184	0.001	1.899
*Transport*	4,330	8.312	1.089	2.303	12.566
*Structure*	4,330	1.298	0.719	0.187	10.603
*Infra*	4,330	2.704	0.460	0.438	4.096
*Fdi*	4,330	0.017	0.018	0.000	0.132
*Edu*	4,330	4.700	1.131	0.693	7.826

## 4 Empirical analysis

### 4.1 Baseline estimation results

We estimate the average effect of the LCCPS on UEI using DML with a 1:4 sample-splitting scheme. Lasso is used for nuisance-function estimation, and we progressively add linear controls, quadratic terms, and fixed effects. Table 3 shows that the LCCPS coefficient is consistently negative and statistically significant, supporting Hypothesis 1: pilot cities experience lower energy intensity after the policy is implemented.

**Table 3:** Baseline regression results.

	(1)	(2)	(3)	(4)
Var	*UEI*	*UEI*	*UEI*	*UEI*
*Event*	−0.068^***^	−0.056^***^	−0.134^***^	−0.113^***^
	(0.014)	(0.014)	(0.017)	(0.019)
Control (single term)	Y	Y	Y	Y
Control (quadratic term)	N	Y	Y	Y
City FE	N	N	Y	Y
Year FE	N	N	N	Y
N	4330	4330	4330	4330

The dependent variable is urban energy intensity. Column (1) includes linear control terms; Column (2) adds quadratic terms; Columns (3) and (4) additionally include city and year fixed effects, respectively. Robust standard errors are reported in parentheses. ^***^p < 0.01.

### 4.2 Robustness checks

#### 4.2.1 Add new fixed effects

To further address time-varying confounding, we augment the baseline with finer-grained fixed effects. Specifically, we add city-specific time trends (city–year effects) and province–year effects to capture shared shocks within provinces. As shown in Table 4 (columns 1–2), the estimated LCCPS effect remains negative and significant, indicating that the baseline finding is not driven by omitted temporal dynamics.

**Table 4:** Robustness checks I.

	(1)	(2)	(3)	(4)
Var	City-time trends	Province time trends	GFRIPZ	CETP
*Event*	−0.128^***^	−0.105^***^	−0.114^***^	−0.129^***^
	(0.017)	(0.016)	(0.019)	(0.021)
Control (single term)	Y	Y	Y	Y
Control (quadratic term)	Y	Y	Y	Y
City FE	Y	Y	Y	Y
Year FE	Y	Y	Y	Y
N	4,330	4,330	4,330	4,330

The dependent variable is urban energy intensity. Columns (1)–(2) report models with additional fixed effects; Columns (3)–(4) additionally control for the Green Finance Reform and Innovation Pilot Zone (GFRIPZ) and Carbon Emissions Trading Pilot (CETP), respectively. Robust standard errors are in parentheses. ^***^p < 0.01.

#### 4.2.2 Exclude the impact of other policies

Because multiple environmental policies overlapped during the sample period, we test whether our estimates are sensitive to concurrent interventions. We control for two closely related national initiatives: the Green Financial Reform and Innovation Pilot Zone (2017) and the Carbon Emissions Trading Pilot (2013). Including these policy indicators leaves the LCCPS estimate largely unchanged (Table 4, columns 3–4), suggesting that our results are not driven by policy co-movements.

#### 4.2.3 Reset the DML model

We also assess robustness to key DML design choices. First, we vary the sample-splitting ratio (1:2 and 1:7) to check sensitivity to the amount of training data. Second, we swap the nuisance estimators to include random forests, gradient boosting, and ordinary least squares. Third, we estimate a more flexible interactive model. These alternatives yield consistently negative policy effects (Table 5), reinforcing the stability of the main conclusion.

**Table 5:** Robustness checks II.

	(1)	(2)	(3)	(4)	(5)	(6)
Var	Kfolds = 3	Kfolds = 8	Rf	Gradboost	Ols	Interactive model
*Event*	−0.108^***^	−0.108^***^	−0.053^***^	−0.070^***^	−0.121^***^	−0.0459^***^
	(0.019)	(0.018)	(0.018)	(0.013)	(0.017)	(0.009)
Control (single term)	Y	Y	Y	Y	Y	Y
Control (quadratic term)	Y	Y	Y	Y	Y	Y
City FE	Y	Y	Y	Y	Y	Y
Year FE	Y	Y	Y	Y	Y	Y
N	4,330	4,330	4,330	4,330	4,330	4,330

The dependent variable is urban energy intensity. Columns (1)–(2) vary the sample split ratio; Columns (3)–(5) use random forest, gradient boosting, and elastic net for prediction; Column (6) estimates an interactive model. Robust standard errors are in parentheses. ^***^p < 0.01.

Across columns (1)–(6) of Table 5, changes in K-fold splitting, machine-learning algorithms, or model form do not overturn the estimated UEI-reducing effect of the LCCPS; effect sizes vary modestly, as expected.

#### 4.2.4 Adjust the research sample

Municipalities directly under the central government (Beijing, Tianjin, Shanghai, Chongqing) differ from other cities in scale and governance. Excluding these municipalities does not change our qualitative finding: the LCCPS estimate remains negative and significant (Table 6, column 1).

**Table 6:** Robustness checks III.

	(1)	(2)	(3)	(4)
Var	Delete center city	5%	PLIV	IIVM
*Event*	−0.115^***^	−0.045^***^	−0.559^**^	−0.743^**^
	(0.018)	(0.012)	(0.394)	(0.472)
Control (single term)	Y	Y	Y	Y
Control (quadratic term)	Y	Y	Y	Y
City FE	Y	Y	Y	Y
Year FE	Y	Y	Y	Y
N	4,330	4,330	4,330	4,330

The dependent variable is urban energy intensity. Column (1) excludes municipalities; Column (2) applies 5% winsorization; Columns (3)–(4) report IV-DML estimates using the partially linear IV model (PLIV) and the interactive IV model (IIVM). Robust standard errors are in parentheses. ^***^p < 0.01, ^**^p < 0.05.

#### 4.2.5 Elimination of outliers

We further test sensitivity to outlier handling by winsorizing continuous variables at the 5th and 95th percentiles. The LCCPS coefficient remains significantly negative (Table 6, column 2).

#### 4.2.6 Instrumental variable

Pilot selection may be non-random if it correlates with city characteristics such as development level and industrial structure. To address this concern, we estimate a partially linear instrumental-variable DML model following Bach et al. (45).


$$\begin{array}{l} UEI_{it}=\theta_{0}Event_{it}+g\left(X_{it}\right)+U_{it} \end{array}$$ (17)

$$\begin{array}{l} IV_{it}=m\left(X_{it}\right)+V_{it} \end{array}$$ (18)

Building on Cai et al. (46), we use the air flow (ventilation) coefficient as an instrument. The coefficient is determined by meteorological conditions (wind speed and boundary-layer height), supporting exogeneity (47). It also affects the likelihood of stricter regulation and thus pilot designation because poorer dispersion can raise the perceived need for stronger environmental governance (48). The IV-DML estimates remain negative and statistically significant (Table 6, columns 3–4), consistent with our baseline results.

## 5 Channel analysis

Having established that the LCCPS reduces UEI on average, we next examine two mechanisms consistent with the policy design. At the macro level, fiscal support can steer investment toward low-carbon infrastructure and facilitate industrial upgrading. At the micro level, enterprise green innovation can improve production efficiency and reduce energy use per unit of output. Together, these channels provide a coherent pathway from policy implementation to lower UEI.

### 5.1 Financial support

Following Liu (49), we proxy fiscal support intensity as the ratio of total fixed-asset investment to local general budgetary expenditure (CNRDS). Table 7 (column 1) shows that the LCCPS significantly increases this indicator, suggesting stronger fiscal guidance toward investment after pilot adoption.

**Table 7:** Channel analysis.

	(1)	(2)
Var	Financial support	Technical investment
*Event*	0.011^**^	0.273^***^
	(0.005)	(0.077)
Control (single term)	Y	Y
Control (quadratic term)	Y	Y
City FE	Y	Y
Year FE	Y	Y
N	4,330	4,330

Column (1) reports the effect of the LCCPS on fiscal support intensity; Column (2) reports the effect on green technological innovation. Robust standard errors are in parentheses. ^***^p < 0.01, ^**^p < 0.05.

A higher fiscal-support intensity can plausibly reduce UEI by redirecting public and private capital toward energy-saving infrastructure and cleaner industrial projects, thereby improving the efficiency of the urban economic system.

### 5.2 Technical investment

For technological investment, we measure green innovation using the logarithm of green invention patent applications following Zhao et al. (50) (CNRDS). Table 7 (column 2) indicates that the LCCPS significantly raises green patenting activity in pilot cities.

Green innovation can lower UEI by enabling cleaner processes, improving energy management, and facilitating substitution away from high-carbon technologies. The results are consistent with an innovation channel that supports sustained energy-intensity reductions.

## 6 Heterogeneity test

### 6.1 Subgroup analysis

We assess whether the LCCPS effect varies across city characteristics using grouped DML with random forests. This approach allows flexible heterogeneity analysis while remaining consistent with the causal-forest framework used below.

#### 6.1.1 Geographical location

City scale may shape policy capacity, industrial composition, and the scope for efficiency gains. Using the National Bureau of Statistics classification, we split the sample into large cities and small/medium cities. Table 8 (columns 1–2) shows a significant UEI reduction in large cities, while the effect in small/medium cities is statistically indistinguishable from zero.

**Table 8:** Heterogeneity test results.

	(1)	(2)	(3)	(4)	(5)	(6)
Var	Small to medium	Large	Inland	Coastal	Lack	Affluence
*Event*	0.011	−0.082^***^	−0.009	−0.060^***^	−0.034^**^	−0.015
	(0.019)	(0.018)	(0.016)	(0.022)	(0.023)	(0.022)
Control (single term)	Y	Y	Y	Y	Y	Y
Control (quadratic term)	Y	Y	Y	Y	Y	Y
City FE	Y	Y	Y	Y	Y	Y
Year FE	Y	Y	Y	Y	Y	Y
N	4,330	4,330	4,330	4,330	4,330	4,330

The dependent variable is urban energy intensity. Robust standard errors are in parentheses. ^***^p < 0.01, ^**^p < 0.05.

A plausible interpretation is that large cities typically have stronger fiscal capacity, deeper innovation ecosystems, and more mature governance systems, which can translate policy signals into measurable efficiency gains. Smaller cities may face tighter budget constraints, weaker innovation bases, and greater dependence on energy-intensive industries, limiting short-run responsiveness.

#### 6.1.2 Geographical location

Coastal and inland cities differ in development level, openness, and energy structures. Following the China Marine Statistical Yearbook classification, we define 108 coastal cities and 165 inland cities. The LCCPS effect is significant in coastal areas but not in inland areas (Table 8, columns 3–4).

Coastal cities may benefit from higher openness and easier access to advanced low-carbon technologies and management practices, as well as stronger administrative capacity. Inland cities, which often remain in energy-intensive industrialization phases, may require complementary support to overcome structural and financial constraints on transition.

#### 6.1.3 Energy endowment

Resource endowment can create different transition constraints. Using the China Coal Industry Yearbook classification, we separate energy-rich and energy-poor cities. Table 8 (columns 5–6) indicates that the LCCPS reduces UEI in energy-poor cities, while the effect in energy-rich cities is not statistically significant.

Energy-rich cities may face stronger lock-in to extraction and heavy-industry pathways, making structural change more costly and politically challenging in the short term. By contrast, resource-scarce cities have stronger incentives to pursue efficiency and diversification, which may amplify the pilot effect.

### 6.2 Causal forests

To further characterize treatment-effect heterogeneity beyond pre-defined groups, we estimate causal forests. Unlike grouped regressions, causal forests can uncover continuous variation in effects and generate city-level conditional average treatment effects (CATEs) (51, 52). We implement the generalized random forest approach using the CausalForestDML function in EconML, selecting key hyperparameters via cross-validation.

**Figure 2:**
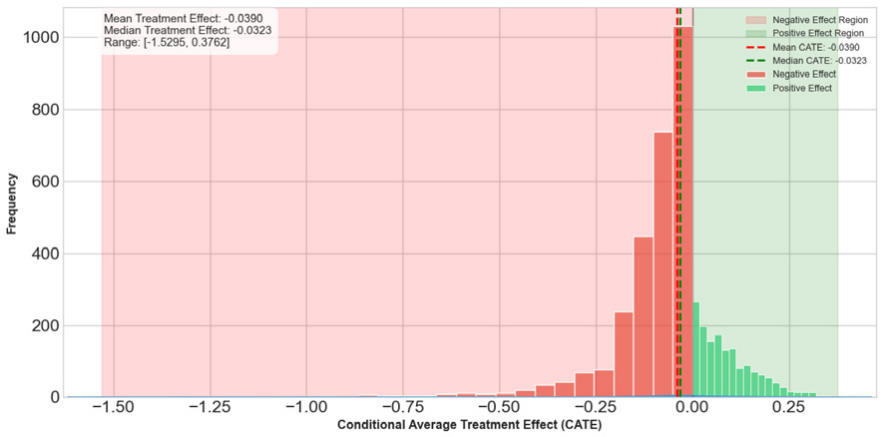
Distribution of conditional average treatment effects.

Figure 2 plots the distribution of estimated CATEs. The average effect is negative, indicating that most cities experience UEI reductions after pilot adoption, although a small number of cities show weak or even positive estimated effects, consistent with meaningful heterogeneity in implementation and local conditions.

**Figure 3:**
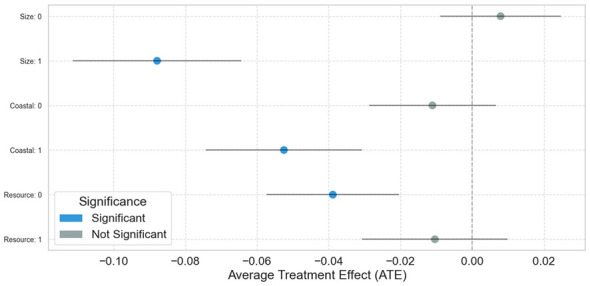
Conditional average treatment effects by different dimensions.

In the heterogeneity test (as shown in Figure 3), this study conducted grouped regression analysis from three dimensions: city size, geographical location, and resource endowment to explore the differential effects of LCCPS implementation. As shown in the figure, “0” represents small and medium-sized cities (Size: 0), inland areas (Coastal: 0), and resource-poor regions (Resource: 0), while “1” represents large cities (Size: 1), coastal areas (Coastal: 1), and resource-rich regions (Resource: 1). Blue dots indicate statistically significant effects, while gray dots represent statistically non-significant effects. The research results show that the LCCPS has a significant reducing effect on energy intensity in large cities, coastal areas, and resource-poor regions; while its suppression effect on energy intensity is not significant in small and medium-sized cities, inland areas, and resource-rich regions. When using causal forest for heterogeneity analysis, the model also identified these three variables as important feature variables, and its results are consistent with the conclusions of the grouped regression. The figure shows the Average Treatment Effect for different groups, further proving that the policy's reduction effect is more significant in larger cities, coastal areas, and resource-poor cities, as indicated by the blue dots representing statistically significant effects. This mutually confirms the results of the grouped regression, enhancing the robustness of the conclusions.

## 7 Conclusions and policy recommendations

### 7.1 Conclusions

This study evaluates the Low-Carbon City Pilot Scheme as a place-based instrument for improving urban energy efficiency and supporting cleaner urban environments. Using a DML framework on panel data for 273 Chinese cities (2006–2021), we find that the LCCPS significantly reduces urban energy intensity. The evidence also supports two pathways—stronger fiscal support and increased enterprise green innovation—and reveals substantial heterogeneity, with larger effects in large cities, coastal areas, and resource-scarce regions. Overall, the results suggest that strengthening low-carbon city governance can help decouple growth from energy use and may generate public-health co-benefits by reducing energy-related environmental pressures.

### 7.2 Policy recommendations

#### 7.2.1 Strengthening fiscal and innovation support for low-carbon cities

Low-carbon city pilots appear capable of lowering UEI, which can plausibly support public-health co-benefits through cleaner air and healthier urban environments. To strengthen impacts, cities can pair clear fiscal guidance with innovation incentives: establish dedicated low-carbon funds, prioritize clean energy/heating and low-carbon transport projects, and use targeted tax relief or subsidies to reduce firms' innovation costs. Regional green-technology alliances linking governments, firms, and research institutions can also speed the diffusion of energy-saving technologies.

#### 7.2.2 Tailored low-carbon pathways and regional coordination

Because impacts differ across city types, policy packages should be more place-specific. Large and coastal cities can scale demonstration programs (e.g., zero-carbon industrial parks, green mobility systems, and building retrofits) and strengthen routine energy–environment monitoring. Resource-scarce cities may gain from faster structural upgrading toward services and cleaner energy substitution. In small/medium, inland, and resource-rich cities, complementary tools—targeted transfers, technical assistance, and transition finance—may be needed to overcome reliance on energy-intensive industries and to ensure that environmental and health gains are shared more equitably.

#### 7.2.3 Strengthening urban energy governance and stakeholder engagement

Improving urban energy governance can consolidate these gains. Integrated platforms that track energy use, emissions, and environmental indicators can support evidence-based management and accountability. From a public-health perspective, incorporating air-quality metrics and vulnerability mapping into performance evaluation can better align decarbonization with health protection. Strengthening corporate disclosure on energy use and emissions, together with multi-stakeholder participation (government, firms, researchers, communities), can improve transparency and sustain long-run reductions in UEI.

### 7.3 Limitations and future research

This study treats UEI as an upstream determinant of emissions and environmental conditions but does not directly quantify downstream health outcomes (e.g., air-pollution exposure, morbidity, or mortality). Future work could link city-level UEI changes to air-quality and health datasets to estimate the full chain of co-benefits and distributional impacts. Exploring spillovers and potential relocation effects with spatial causal methods would further inform the design of low-carbon policies that are both effective and health-equitable.

## Data Availability

The raw data supporting the conclusions of this article will be made available by the authors, without undue reservation.

## References

[1] Lu Z Shao C Wang F Dong R. Evaluation of green and low-carbon development level of chinese provinces based on sustainable development goals. Sustainability. (2023) 15:15449. doi: 10.3390/su152115449

[2] Zeng N Jiang K Han P Hausfather Z Cao J Kirk-Davidoff D . The Chinese carbon-neutral goal: challenges and prospects. Adv Atmos Sci. (2022) 39:1229–38. doi: 10.1007/s00376-021-1313-6 35095159 PMC8787441

[3] Wang M Guo B. Have industrial robots reduced carbon emissions? Empirical evidence from China. Technol Soc. (2026) 86:103244. doi: 10.1016/j.techsoc.2026.103244

[4] Shan Y Guan Y Hang Y Zheng H Li Y Guan D . City-level emission peak and drivers in China. Sci Bull. (2022) 67:1910–20. doi: 10.1016/j.scib.2022.08.024 36546305

[5] Ali U Guo Q Kartal MT Nurgazina Z Khan ZA Sharif A. The impact of renewable and non-renewable energy consumption on carbon emission intensity in China: fresh evidence from novel dynamic ARDL simulations. J Environ Manage. (2022) 320:115782. doi: 10.1016/j.jenvman.2022.115782 35963066

[6] Guo B Li M. Does the application of industrial robots enhance urban energy resilience? Evidence from China. Energies. (2026) 19:1555. doi: 10.3390/en19061555

[7] Wang J Zhang S Zhang Q. The relationship of renewable energy consumption to financial development and economic growth in China. Renew Energy. (2021) 170:897–904. doi: 10.1016/j.renene.2021.02.038

[8] Wang W Liu S Guo B. Impact of national health city campaign on public health in China. Front Public Health. (2025) 13:1594104. doi: 10.3389/fpubh.2025.1594104 40376053 PMC12078279

[9] Mao F Lin J Hou Y. Dynamic evolution and frontiers of energy decentralization research: a bibliometric-based review. Energy Strategy Rev. (2026) 64:102097. doi: 10.1016/j.esr.2026.102097

[10] Jiang Y Zhang Z Xie G. Emission reduction effects of vertical environmental regulation: capacity transfer or energy intensity reduction? Evidence from a quasi-natural experiment in China. J Environ Manage. (2022) 323:116180. doi: 10.1016/j.jenvman.2022.116180 36103792

[11] Du K Cheng Y Yao X. Environmental regulation, green technology innovation, and industrial structure upgrading: the road to the green transformation of Chinese Cities. Energy Econ. (2021) 98:105247–105247. doi: 10.1016/j.eneco.2021.105247

[12] Fu Y He C Luo L. Does the low-carbon city policy make a difference? Empirical evidence of the pilot scheme in China with DEA and PSM-DID. Ecol Indic. (2021) 122:107238. doi: 10.1016/j.ecolind.2020.107238

[13] Feng T Lin Z Du H Qiu Y Zuo J. Does low-carbon pilot city program reduce carbon intensity? Evidence from Chinese Cities. Res Int Bus Finance. (2021) 58:101450. doi: 10.1016/j.ribaf.2021.101450

[14] Wang S Guo B. Impact of green finance on urban ecological and environmental resilience: evidence from China. Sustainability. (2026) 18:706. doi: 10.3390/su18020706

[15] Jiang J Lin J Guo B. How does digital-green policy synergy affect substantive and strategic green technology innovation? Evidence from China. Int Rev Econ Finance. (2026) 106:104969. doi: 10.1016/j.iref.2026.104969

[16] Gao D Li Y Li G. Boosting the green total factor energy efficiency in Urban China: does low-carbon city policy matter? Environ Sci Pollut Res Int. (2022) 29:56341–56356. doi: 10.1007/s11356-022-19553-9 35334053

[17] Yu Y Zhang N. Low-carbon city pilot and carbon emission efficiency: quasi-experimental evidence from China. Energy Econ. (2021) 96:105125. doi: 10.1016/j.eneco.2021.105125

[18] Du M Feng R Chen Z. Blue sky defense in low-carbon pilot cities: a spatial spillover perspective of carbon emission efficiency. Sci Total Environ. (2022) 846:157509. doi: 10.1016/j.scitotenv.2022.157509 35870596

[19] Knittel CR Stolper S. Machine learning about treatment effect heterogeneity: the case of household energy use. AEA Pap Proc. (2021) 111:440–4. doi: 10.1257/pandp.20211090

[20] Aiken E Bellue S Karlan D Udry C Blumenstock JE. Machine learning and phone data can improve targeting of humanitarian aid. Nature. (2022) 603:864–870. doi: 10.1038/s41586-022-04484-9 35296856 PMC8967719

[21] Yan MR Yan H Chen YR Zhang Y Yan X Zhao Y. Integrated green supply chain system development with digital transformation. Int J Logist Res Appl. (2026) 29:394–415. doi: 10.1080/13675567.2025.2492217

[22] Macikenaite V. China's economic statecraft: the use of economic power in an interdependent world. J Contemp East Asia Stud. (2020) 9:108–126. doi: 10.1080/24761028.2020.1848381

[23] Li K Zhou Y Xiao H Li Z Shan Y. Decoupling of economic growth from co2 emissions in Yangtze River economic belt cities. Sci Total Environ. (2021) 775:145927. doi: 10.1016/j.scitotenv.2021.145927

[24] Zeng S Jin G Tan K Liu X. Can low-carbon city construction reduce carbon intensity? Empirical evidence from low-carbon city pilot policy in China. J Environ Manage. (2023) 332:117363. doi: 10.1016/j.jenvman.2023.117363 36736083

[25] Peng B Wang Y Wei G. Energy eco-efficiency: is there any spatial correlation between different regions? Energy Policy. (2020) 140:111404. doi: 10.1016/j.enpol.2020.111404

[26] Liu X Xu H. Does low-carbon pilot city policy induce low-carbon choices in residents' living: holistic and single dual perspective. J Environ Manage. (2022) 324:116353. doi: 10.1016/j.jenvman.2022.116353 36182842

[27] Ren H Gu G Zhou H. Assessing the low-carbon city pilot policy on carbon emission from consumption and production in china: how underlying mechanism and spatial spillover effect? Environ Sci Pollut Res Int. (2022) 29:71958–71977. doi: 10.1007/s11356-022-21005-3 35610453

[28] Song M Zhao X Shang Y. The impact of low-carbon city construction on ecological efficiency: empirical evidence from quasi-natural experiments. Resour Conserv Recycl. (2020) 157:104777. doi: 10.1016/j.resconrec.2020.104777

[29] Guan H Zhang Y Zhao A. Environmental taxes, enterprise innovation, and environmental total factor productivity-effect test based on porter's hypothesis. Environ Sci Pollut Res Int. (2023) 30:99885–99. doi: 10.1007/s11356-023-29407-7 37620703

[30] Farghali M Osman AI Mohamed I.M.A, Chen Z, Chen L, Ihara I, et al. Strategies to save energy in the context of the energy crisis: a review. Environ Chem Lett. (2023) 21:2003–2039. doi: 10.1007/s10311-023-01591-5 PMC10035500 37362011

[31] Tang K Liu Y Zhou D Qiu Y. Urban carbon emission intensity under emission trading system in a developing economy: evidence from 273 Chinese Cities. Environ Sci Pollut Res Int. (2020) 28:5168–5179. doi: 10.1007/s11356-020-10785-1 32959321

[32] Zhang J Chu Z Sun Z. Impact of low-carbon city pilot policies on urban green innovation from the perspective of spatial and temporal heterogeneity. Environ Sci Pollut Res Int. (2023) 30:114358–114374. doi: 10.1007/s11356-023-30320-2 37861828

[33] Chen H Guo W Feng X Wei W Liu H Feng Y . The impact of low-carbon city pilot policy on the total factor productivity of listed enterprises in China. Resour Conserv Recycl. (2021) 169:105457. doi: 10.1016/j.resconrec.2021.105457

[34] Guo X Song X Dou B Wang A Hu H. Can digital transformation of the enterprise break the monopoly? Pers Ubiquit Comput. (2022) 27:1629–42. doi: 10.1007/s00779-022-01666-0

[35] Miao CL Meng XN Duan MM Wu XY. Energy consumption, environmental pollution, and technological innovation efficiency: taking industrial enterprises in China as Empirical Analysis Object. Environ Sci Pollut Res Int. (2020) 27:34147–34157. doi: 10.1007/s11356-020-09537-y 32557046

[36] Liu C Ma C Xie R. Structural, innovation and efficiency effects of environmental regulation: evidence from China's Carbon Emissions Trading Pilot. Environ Resource Econ. (2020) 75:741–768. doi: 10.1007/s10640-020-00406-3

[37] Li W Fan J Zhao J. Has green finance facilitated China's low-carbon economic transition? Environ Sci Pollut Res Int. (2022) 29:57502–15. doi: 10.1007/s11356-022-19891-8 35353311

[38] Xia W Zheng S Qiu L Hu H Chen Y Wei S . Evolution of the innovation network of lithium-ion battery recycling technologies in China from the perspective of patents. Pol J Environ Stud. (2026) 35:2903–16. doi: 10.15244/pjoes/203046

[39] Lyu J Liu T Cai B Qi Y Zhang X. Heterogeneous effects of China's low-carbon city pilots policy. J Environ Manage. (2023) 344:118329. doi: 10.1016/j.jenvman.2023.118329 37379627

[40] He Y Lin B. Investigating environmental Kuznets curve from an energy intensity perspective: empirical evidence from China. J Clean Prod. (2019) 234:1013–22. doi: 10.1016/j.jclepro.2019.06.121

[41] Badulescu D Badulescu A Simut R Bac D Iancu EA Iancu N. EXPLORING ENVIRONMENTAL KUZNETS CURVE. AN INVESTIGATION ON EU ECONOMIES. Technol Econ Dev Econ. (2019) 26:1–20. doi: 10.3846/tede.2019.11261

[42] Chernozhukov V Chetverikov D Demirer M Duflo E Hansen C Newey W . Double/debiased machine learning for treatment and structural parameters. Econom J. (2018) 21:C1–C68. doi: 10.1111/ectj.12097

[43] Yang Z Wei X. The measurement and influences of China's urban total factor energy efficiency under environmental pollution: based on the game cross-efficiency DEA. J Clean Prod. (2019) 209:439–50. doi: 10.1016/j.jclepro.2018.10.271

[44] Li M Liu T Wu G Lin J Guo B. Has the development of artificial intelligence promoted urban pollutant and carbon emission reduction? Evidence from China. Front Public Health. (2026) 13:1739342. doi: 10.3389/fpubh.2025.1739342 41607909 PMC12835399

[45] Bach P Kurz MS Chernozhukov V Spindler M Klaassen S. DoubleML: an object-oriented implementation of double machine learning in R. J Stat Soft. (2024) 108. doi: 10.18637/jss.v108.i03

[46] Cai H Wang Z Zhang Z Xu L. Does environmental regulation promote technology transfer? Evidence from a partially linear functional-coefficient panel model. Econ Model. (2023) 124:106297. doi: 10.1016/j.econmod.2023.106297

[47] Huang L Lei Z. How environmental regulation affect corporate green investment: evidence from China. J Clean Prod. (2021) 279:123560. doi: 10.1016/j.jclepro.2020.123560

[48] Li Z Lai A Cao Y Wang Q. Porter effect vs cost effect: the impact of China's low carbon city pilot on carbon emissions and economic performance. J Environ Manage. (2024) 360:121015. doi: 10.1016/j.jenvman.2024.121015 38744209

[49] Liu C. Infrastructure public–private partnership (PPP) investment and government fiscal expenditure on science and technology from the perspective of sustainability. Sustainability. (2021) 13:6193. doi: 10.3390/su13116193

[50] Zhao N Liu X Pan C Wang C. The performance of green innovation: from an efficiency perspective. Socioecon Plann Sci. (2021) 78:101062. doi: 10.1016/j.seps.2021.101062

[51] Wager S Athey S. Estimation and inference of heterogeneous treatment effects using random forests. J Am Stat Assoc. (2018) 113:1228–42. doi: 10.1080/01621459.2017.1319839

[52] Li ZF Zhou Q Chen M Liu Q. The impact of COVID-19 on industry-related characteristics and risk contagion. Financ Res Lett. (2021) 39:101931. doi: 10.1016/j.frl.2021.101931 33519308 PMC7834769

